# Conversion of H_2_ and CO_2_ to CH_4_ and acetate in fed-batch biogas reactors by mixed biogas community: a novel route for the power-to-gas concept

**DOI:** 10.1186/s13068-016-0515-0

**Published:** 2016-05-10

**Authors:** Márk Szuhaj, Norbert Ács, Roland Tengölics, Attila Bodor, Gábor Rákhely, Kornél L. Kovács, Zoltán Bagi

**Affiliations:** Department of Biotechnology, University of Szeged, Közép fasor 52, Szeged, 6726 Hungary; Institute of Biochemistry, Biological Research Center, Hungarian Academy of Sciences, Temesvári krt. 62, Szeged, 6726 Hungary; Department of Oral Biology and Experimental Dental Research, University of Szeged, Tisza L. krt. 64, Szeged, 6720 Hungary; Institute of Biophysics, Biological Research Centre, Hungarian Academy of Sciences, Temesvári krt. 62, Szeged, 6726 Hungary

**Keywords:** Biomethane, Hydrogen, Carbon dioxide, Hydrogenotrophic methanogens, Power-to-gas, Power-to-biomethane (P2B)

## Abstract

**Background:**

Applications of the power-to-gas principle for the handling of surplus renewable electricity have been proposed. The feasibility of using hydrogenotrophic methanogens as CH_4_ generating catalysts has been demonstrated. Laboratory and scale-up experiments have corroborated the benefits of the CO_2_ mitigation via biotechnological conversion of H_2_ and CO_2_ to CH_4_. A major bottleneck in the process is the gas–liquid mass transfer of H_2_.

**Results:**

Fed-batch reactor configuration was tested at mesophilic temperature in laboratory experiments in order to improve the contact time and H_2_ mass transfer between the gas and liquid phases. Effluent from an industrial biogas facility served as biocatalyst. The bicarbonate content of the effluent was depleted after some time, but the addition of stoichiometric CO_2_ sustained H_2_ conversion for an extended period of time and prevented a pH shift. The microbial community generated biogas from the added α-cellulose substrate with concomitant H_2_ conversion, but the organic substrate did not facilitate H_2_ consumption. Fed-batch operational mode allowed a fourfold increase in volumetric H_2_ load and a 6.5-fold augmentation of the CH_4_ formation rate relative to the CSTR reactor configuration. Acetate was the major by-product of the reaction.

**Conclusions:**

Fed-batch reactors significantly improve the efficiency of the biological power-to-gas process. Besides their storage function, biogas fermentation effluent reservoirs can serve as large-scale bio CH_4_ reactors. On the basis of this recognition, a novel concept is proposed, which merges biogas technology with other means of renewable electricity production for improved efficiency and sustainability.

## Background

Pressing deterioration of the global climate by human activities demands the large-scale replacement of fossil fuels with renewable energy carriers [1]. The most rapidly developing and spreading renewable technologies worldwide include the conversion of wind energy and direct solar energy (photovoltaics) to electricity. In view of the discontinuous electricity production by these technologies, coupled with fluctuating utilization, severe electricity storage problems arise, which are already apparent in countries where the implementation of renewables is well advanced. A likely solution of this emerging setback is conversion of electricity to alternative energy carriers [2] or chemicals [3]. Hydrogen (H_2_) can be generated via electrolysis of water, a well-known and efficient process [4]; however, technologies to store and transport H_2_ are underdeveloped at present. Methane (CH_4_) is an obvious next candidate. CH_4_ can be transported and stored conveniently in the existing natural gas grid and can be used in all applications where fossil natural gas is employed today. Biogenic CH_4_ production takes place during anaerobic degradation of organic matter in biogas reactors, swamps, ruminants, termites, etc. [2]. The last step of these complex microbiological metabolic pathways is biogas formation by methanogens. These microbes are strict anaerobes belonging in the kingdom Archaea. Some methanogens split acetate and release a mixture of CH_4_ and CO_2_ (acetotrophic methanogens), while others form CH_4_ by reducing CO_2_ with H_2_ (hydrogenotrophic methanogens) and there are methanogens which are able to carry out both reactions.

An additional advantage of the biological conversion of electricity to CH_4_ is offered by coupling the process with CO_2_ mitigation. CO_2_ can be transformed by catalytic reduction using chemical reactions [5, 6], photosynthesis [7], bioelectrochemical processes [8–10], or methanogenesis [2].

Three main ingredients should be present to form biogenic CH_4_ from CO_2_: hydrogenotrophic methanogens, CO_2_, and a suitable reductant. Recent metagenomic studies have revealed that hydrogenotrophic methanogens predominate among Archaea in most biogas microbial communities [11–17].

CO_2_ can originate from flue gas [18] or from the biogas itself [19–22]. In the latter approach, a significant upgrading of the produced biogas has been achieved. In some cases, the anaerobic degradation of the biomass has provided the electron source [18, 23]; in other studies, H_2_ gas has been employed [19, 20, 22, 24]. These experiments have revealed that the poor solubility of H_2_ limits the yield and rate of CH_4_ formation. In these configurations, H_2_ is injected into a methanogenic reactor filled with a microbial consortium.

In the present study, fed-batch fermentation systems with the daily dispensing of H_2_ gas were employed in order to partially overcome the H_2_ solubility problem. Several operational conditions (see “Methods” section) were tested under mesophilic conditions and efficient CH_4_ productivity was attained. Moreover, at the appropriate combination of CO_2_ and H_2_, the simultaneous formation of acetate and CH_4_ as main products was observed.

## Results

### Effect of mixing

**Fig. 1:**
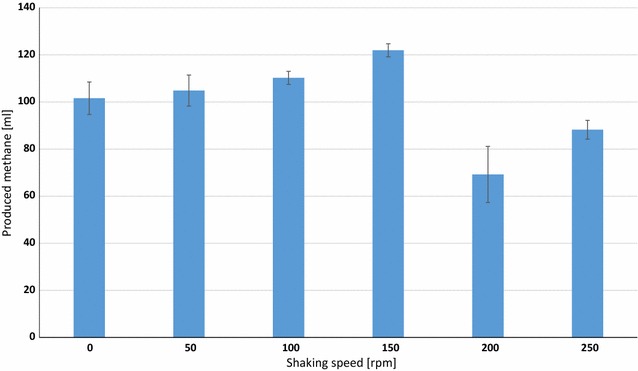
Dependence of the CH_4_ production on shaking speed

Given the experimental conditions (see “Methods” section) and the poor solubility of H_2_ in the aqueous phase, the optimal mixing conditions yielding the most efficient delivery of H_2_ from the gas phase had to be determined. The reaction vessels were incubated in an orbital shaker at various mixing speeds (rpm). Figure 1 indicates that there is an optimum value for this parameter; in our arrangement, it was 150–160 rpm. It is noteworthy that at higher mixing rates CH_4_ production decreased sharply in contrast to earlier observations at thermophilic temperature [19]. In all subsequent experiments, the shaker was set at 160 rpm. It is evident that this mixing rate is valid under our conditions and henceforth was applied consistently in order to limit the number varying parameters. In other systems, the optimal mixing conditions should be determined individually. The main conclusion from these experiments was that the mixing that yields optimal H_2_ utilization may not be the maximum achievable mixing rate.

### Optimization of H_2_ dosage

**Fig. 2:**
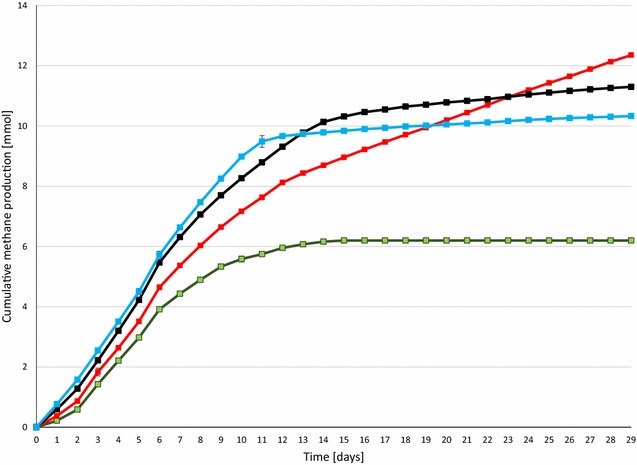
Cumulative CH_4_ production from H_2_. α-cellulose (0.3 g) was added as substrate at the start of the experiment. H_2_ was injected into the reactor headspace daily, following flushing with N_2_. *Green*: no H_2_ added, *red*: 0.79 mmol H_2_ day^−1^, *black* 1.57 mmol H_2_ day^−1^, *blue*: 2.36 mmol H_2_ day^−1^ added

**Fig. 3:**
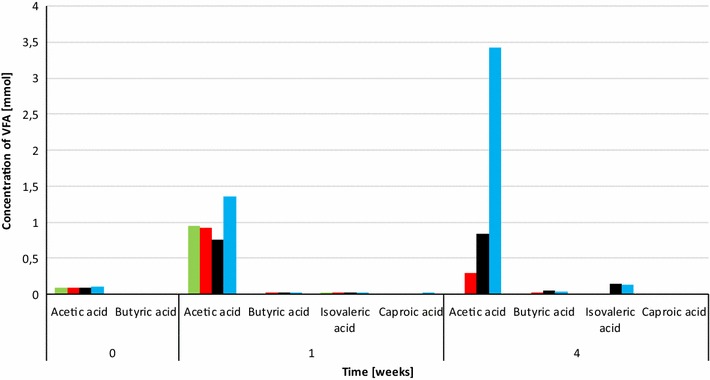
Levels of volatile organic acids in the reactors at the beginning (0 weeks = inoculum and after week 1 and week 4), respectively. The reactors received daily injections of H_2_ gas: 0.0 (*green*), 0.79 (*red*), 1.57 (*black*), and 2.36 (*blue*)

**Fig. 4:**
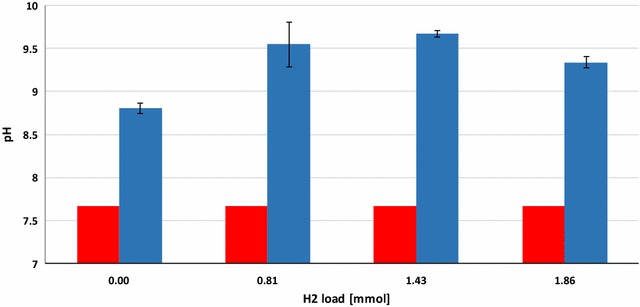
The initial (*red*) and final (*blue*) pH in the liquid phase of the reactors received mmole, respectively. α-cellulose (0.3 g) was added as substrate at the start of the experiment

Next the optimal daily H_2_ dosage was established. Various volumes of H_2_ were therefore injected into the batch reactors, which were treated identically in all other known aspects. The batch fermentations were started by adding 0.3 g of α-cellulose as substrate for AD according to the VDI (Verein Deutscher Ingenieure, protocol [25]. H_2_ gas was injected every day and the consumption of H_2_ was followed by gas chromatography. Cumulative CH_4_ evolution curves are plotted in Fig. 2. CH_4_ production proceeded steadily for 7–8 days in the control reactors, which received no daily H_2_ dosage, but from day 12 practically no gas evolved. In total, 6.2 ± 0.54 mmol of CH_4_ was generated from the residual biogas potential of the sludge and added α-cellulose substrate. 1.62 mmol of this quantity originated from the sludge and 4.58 mmol from the α-cellulose substrate. The biochemical CH_4_ potential of α-cellulose is 4.71 [26] and therefore all of the added substrate was consumed by the community and was converted to CH_4_. Addition of a daily 0.81 ± 0.16 mmol of H_2_ gas into the headspace of the batch reactors dramatically increased the CH_4_ production (Fig. 2). The GC measurements revealed that all of the injected H_2_ was completely consumed by the microbes within 24 h. In separate experiments, it was established more precisely that under these conditions all the H_2_ had vanished from the headspace after 16 h and CH_4_ evolution started at hour 2 following H_2_ injection (data not shown). A new dosage of H_2_ was dispensed consistently every 24 h. Increasing the total H_2_ load to 43.00 ± 1.43 mmol resulted in a somewhat faster initial CH_4_ production, but the cumulative-specific CH_4_ production was lower than in the case of adding 24.42 ± 0.81 mmol of H_2_ in the same period of time. In line with this observation, H_2_ started to accumulate in the headspace on day 14 and from day 17–18 CH_4_ production ceased. On further increase of the overall H_2_ injection volume to 55.69 ± 1.85 mmol, i.e., 1.86 ± 0.38 mmol H_2_ day^−1^, even less cumulative-specific CH_4_ was yielded. In these reactors, H_2_ build-up in the headspace started sooner, i.e., on day 10 and CH_4_ evolution stopped completely on day 13. Overall, these results indicated that the system utilized the α-cellulose substrate within 7–8 days and the microbial community sustained its H_2_ conversion activity for an extended period of time if the daily H_2_ injection did not exceed 0.81 ± 0.16 mmol of H_2_ (Table 1). The concentrations of organic acids were determined every week. Acetate levels increased significantly by the end of the experimental period. 3.43 mM acetate accumulated by the end of the experiment in the reactors receiving 55.69 ± 1.85 mmol of H_2_, which exceeded the recommended threshold, but apparently this alone did not explain why CH_4_ evolution stopped in the reactors loaded with higher daily H_2_ injections (Fig. 3). The pH had increased considerably by the end of the 4-week experiments (Fig. 4), indicating a severe loss of the bicarbonate buffering capacity of the inoculum sludge. It is noteworthy that the pH also shifted by 1.1 units in the control reactors which were not fed with H_2_. In order to employ the same protocol, these vessels were also degassed and filled with N_2_ gas every day. It is therefore likely that the daily replacement of the headspace prompted a gradual desorption and loss of dissolved CO_2_ and caused a shift in the bicarbonate buffering system [27, 28]. The pH increased even further, i.e., beyond pH = 9, which is a critical upper limit for the methanogenesis [29]. A similar exhaustion of the buffering capacity upon H_2_ addition was noted in previous reports [19, 20]. The system could apparently tolerate high pH fairly well when 0.81 ± 0.16 mmol of H_2_ was the daily dosage, but started to inhibit CH_4_ biosynthesis on day 13 and 10 upon addition of daily 1.43 ± 0.28 or 2.86 ± 0.38 mmol of H_2_, respectively. In this experimental set-up, it was not possible to determine the time points when the inhibitory pH range was attained. The results indicated that the likely reason for the obstruction of CH_4_ formation was the limiting buffering capacity of the system due to the low bicarbonate concentration. The optimal amount of daily H_2_ dosage in this system was within the range of 0.8–1.5 mmol of H_2_; further experiments should determine the exact value.

**Table 1:** Origin and balance of CH_4_ formation in the fed-batch reactors supplied with α-cellulose at the start of the experiment and with various amounts of daily H_2_

Total CH_4_ production (mmol)	CH_4_ from α-cellulose (mmol)	Theoretical from α-cellulose (mmol)	Total injected H_2_ (mmol)	Theoretical CH_4_ from H_2_ (mmol)	Measured CH_4_ from H_2_ (mmol)	Difference
6.20 ± 0.54	4.58 ± 0.09	4.71	0.00 ± 0	0.00	0.00 ± 0.66	0.00
12.35 ± 0.44	4.63 ± 0.09	4.71	24.42 ± 0.41	6.10	6.10 ± 0.24	0.00
11.30 ± 0.50	4.61 ± 0.09	4.71	43.00 ± 1.02	10.75	5.08 ± 0.48	−5.67
10.33 ± 0.81	4.61 ± 0.09	4.71	55.69 ± 2.76	13.92	4.11 ± 0.75	−9.82

### Effect of CO_2_ addition

**Fig. 5:**
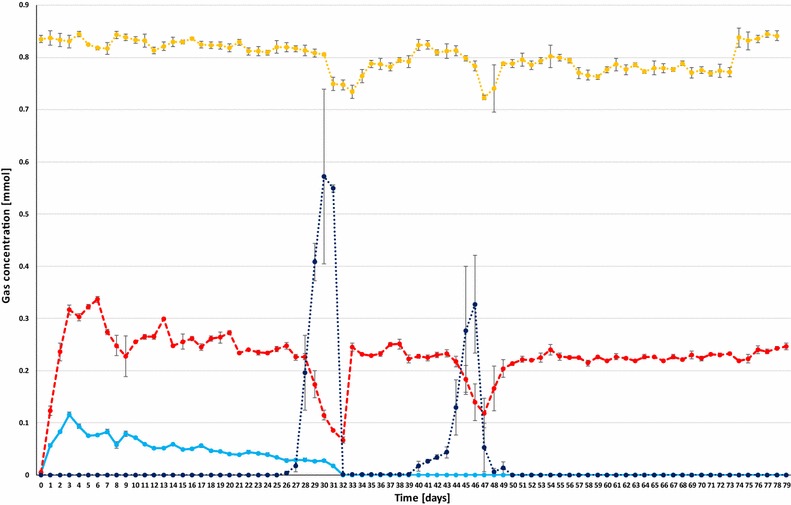
The amount of daily H_2_ injection (*dotted yellow*), CH_4_ production (*red*) and residual H_2_ (*dotted dark blue*). CH_4_ production by the control reactor (no H_2_ injected) is shown in *light blue*. The reactors received no external substrate

**Fig. 6:**
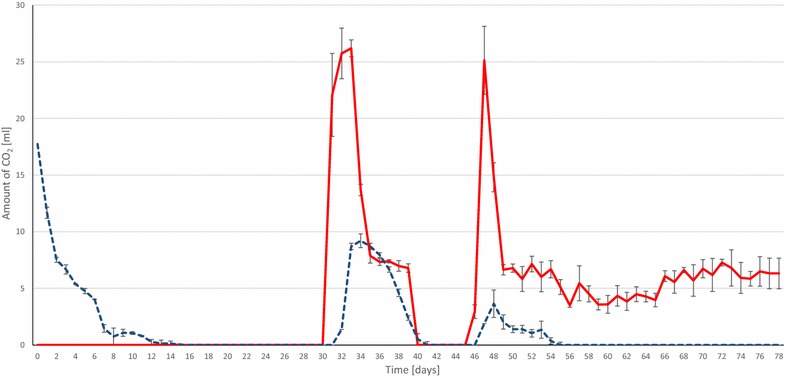
The amount of CO_2_ injected daily (*red*) and the residual CO_2_ concentration in the headspace after 24 h (*dashed blue*). The reactors received no external substrate. Experimental conditions as in Fig. 5

In the next series of batch fermentations, the inoculum originated from the same mesophilic industrial biogas plant, but at different points of time, and therefore small fluctuations of organic total solid content and microbial community composition should be taken into account when the results are subjected to direct comparison. The initial addition of α-cellulose was omitted in order to avoid any disturbing effect of the CH_4_ generation from the substrate. The duration of these fermentations was extended to 80 days to test for sustainable CH_4_ production. The reactors were supplied with the optimal daily dosage of 0.81 mmol of H_2_ in order to check if the CO_2_/bicarbonate buffering capacity was indeed the major limiting factor in the previous experiments [28, 30]. The daily CH_4_ volumes measured in the headspace are plotted in Fig. 5. CH_4_ evolution progressed steadily until day 28, but dropped sharply afterwards. A warning sign of system failure was noticed already on day 27, when measurable residual H_2_ was detected in the headspace (Fig. 5; Table 2). As shock therapy, massive CO_2_ injection (25 mL) was dispensed into the reactors following the daily dosage of H_2_ on day 31 (Fig. 6). All of this CO_2_ disappeared from the gas phase within 24 h, indicating that the system was indeed severely depleted of CO_2_/bicarbonate. The same CO_2_ treatment was repeated next day, which apparently restored the functional state of the system signaled by the build-up of residual CO_2_ in the headspace (Fig. 6). The daily CO_2_ dose was then gradually decreased to the stoichiometric volume, i.e., approximately 0.25 mol of CO_2_/mol of H_2_ per day. The system responded positively, as exhibited by the restoration of CH_4_ production on day 32 accompanied by a gradual decrease of residual CO_2_ levels in the gas phase. Daily CO_2_ injection was stopped on day 41. H_2_ accumulation commenced again almost immediately and was accompanied by the loss of CH_4_-evolving ability from day 43, and therefore CO_2_ injection (25 mL) was resumed on day 47. Detectable remaining CO_2_ was noticed already on the next day and from this time on a daily dosage of 0.25 mol of CO_2_/mol of H_2_ of CO_2_ was maintained until the end of the experiment. CH_4_ production returned to the previous level, all of the injected daily H_2_ and CO_2_ were consumed within 24 h and this continued for an additional month. It is noteworthy that, except for pH bursts on days 31 and 45, the pH in both the control and H_2_-fed reactors remained within the acceptable limit of pH ≤8.5 throughout the investigated period (data not shown).

**Table 2:** Comparison of process parameters between CSTR (Bassani et al. 2015) and fed-batch (present work) bioCH_4_ production approaches

	Bassani et al. (2015)^a^		Present work			
			No external CO_2_		External CO_2_ added	
	Control	H_2_ added	Control	H_2_ added	Control	H_2_ added
Biogas composition (%)						
CH_4_	69.7 ± 0.3	88.9 ± 2.4	17.71 ± 1.15	79.77 ± 2.31	0.00	95.53 ± 1.79
CO_2_	30.3 ± 0.3	8.8 ± 3.2	73.63 ± 3.61	17.71 ± 0.90	0.00	4.47 ± 1.34
H_2_	0	2.3 ± 1.8	0.0	2.51 ± 0.82	0.0	0.00
Gas production (mL L^−1^ h^−1^)						
CH_4_	2.75 ± 0.58	4.17 ± 0.50	1.51 ± 0.07	6.78 ± 0.20	0.00 ± 0.00	6.21 ± 0.12
CH_4_ from H_2_	0.0	1.41	0.00	4.27	0.00	6.21
CO_2_	1.21 ± 0.25	0.42 ± 0.13	4.20 ± 0.21	1.51 ± 0.08	0.00 ± 0.00	0.29 ± 0.09
H_2_ injection rate (mL L^−1^ h^−1^)	0.00	8.00 ± 1.17	0.00^b^	22.66 ± 0.20^b^	0.00^b^	20.96 ± 0.23^b^
H_2_ consumption (mL L^−1^ h^−1^)	0.0	7.42 ± 1.08	0.00 ± 0.00	22.44 ± 0.19	0.00 ± 0.00	20.96 ± 0.02
H_2_ consumption (%)	0.0	92.7	0.0	99.06	0.0	100.00
pH	7.74 ± 0.16	8.17 ± 0.04	8.66 ± 0.19^c^	9.38 ± 0.11^c^	8.29 ± 0.04^c^	7.89 ± 0.20^c^
Organic acids (mM)						
Acetate	nd	nd	nd	nd	0.33	1.48
Butyrate	nd	nd	nd	nd	0.00	0.04
Isovalerate			nd	nd	0.02	0.10

^a^ Mesophilic data

^b^ Estimated from daily dose

^c^ At the end of the experiment; *nd* not determined

Several deductions could be drawn from this series of tests. First, the system becomes depleted of CO_2_ if semi-continuous H_2_ feeding and daily degassing are administered to the fed-batch system. This phenomenon was manifested after about 1 month in our arrangement, where daily degassing and replacement of the headspace were included to retain the same protocol in the control and experimental reactors. Clearly daily degassing is not necessary in industrial setting. Second, the residual H_2_ accumulation in the gas phase is a good early warning sign of upcoming system failure due to CO_2_ exhaustion. Third, the microbial community participating in the CH_4_ generation process recuperates quickly and completely even after repeated system failure if the process control is alerted in time. Fourth, the microbial community supplied only with H_2_ and CO_2_ upholds the pH within the normal operating range. Finally, stoichiometric administration of H_2_ and CO_2_ yields a practically complete conversion to pure CH_4_ within 24 h under mesophilic conditions.

### Effect of additional substrate addition

**Fig. 7:**
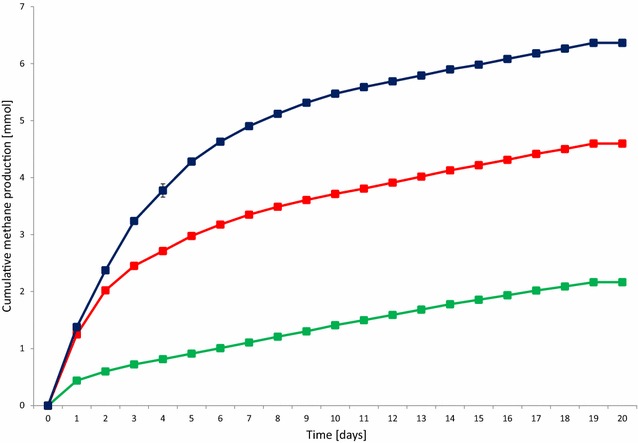
Cumulative CH_4_ production at various initial α-cellulose loadings: 0 g (*green*), 0.3 g (*red*), and 0.6 g (*blue*)

**Fig. 8:**
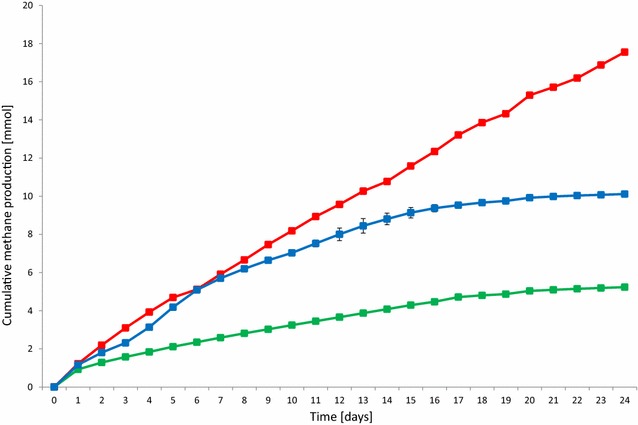
Cumulative CH_4_ production at various weekly α-cellulose loadings: 0 g (*green*), 0.3 g (*red*), and 0.6 g (*blue*)

Next, it was tested whether the addition of α-cellulose affected the CH_4_ productivity from H_2_. Two series of experiments were designed and the duration of the experimental period was shortened in order to avoid any complication due to CO_2_ depletion and concomitant pH elevation. In the first set of batch fermentations (Fig. 7), various amounts of α-cellulose were added only at the start of the experiments, and in the second series (Fig. 8) the addition of the same amount of α-cellulose was repeated every week. Daily replacement of the headspace with N_2_ and the injection of 0.81 mmol of H_2_ was maintained in all reactors.

There was no significant difference between the CH_4_ productions from H_2_ in the reactors receiving the substrate quantity recommended by the VDI [25] protocol as compared with those without substrate, i.e., the difference between the green and red curves in Fig. 7 correspond solely to the CH_4_ produced from α-cellulose. This suggests that the addition of substrate at the beginning of the fermentation does not assist CH_4_ evolution from H_2_. Moreover, an inhibition of CH_4_ productivity from H_2_ was noted when the substrate load was doubled, i.e., upon the addition of 0.6 g substrate, 3.47 ± 0.08 mmol of CH_4_ was formed from α-cellulose instead of the theoretical potential of 9.42 mmol of CH_4_. It should be noted that the H_2_ consumption rate remained unaffected by the substrate loading, i.e., the injected H_2_ disappeared from the headspace within 24 h. The conversion efficiency of CH_4_ formation from H_2_ was estimated from the daily CH_4_ levels in the headspace. The day-to-day values fluctuated considerably during the experimental period and achieved an average of 72 ± 25 %. The remainder of the H_2_ may have been metabolized in alternative pathways, which are the subject of future studies.

In the next set of experiments, the reactors were fed with the same amount (0.3 g) of α-cellulose every week and the daily H_2_ injection (0.81 mol H_2_) was maintained. The aim was to test whether the microbial community remained intact for an extended period of time after the expiration of its residence time in the industrial AD facility and to see whether the metabolically active community facilitated the bioconversion of H_2_ to CH_4_. The cumulative CH_4_ production increased almost linearly and the amount formed suggested an unchanged reaction rate for both α-cellulose and H_2_ when the VDI protocol [25] was followed (Fig. 8). It is noteworthy that increasing the weekly α-cellulose load prompted a strong inhibitory effect. The collapse of the CH_4_-forming activity was not associated with changes in pH. Without α-cellulose, the daily dosage of H_2_ caused an increase of the pH into the dangerous zone, as observed earlier (Fig. 4), due to the depletion of the buffering capacity. Weekly supply of the substrate balanced the pH; the degradation of the α-cellulose apparently yielded enough CO_2_ to maintain stable operation. Too much substrate, e.g., 0.6 g α-cellulose/week, shifted the pH to lower values, although it did not fall below 6.5, which is usually considered detrimental [29]. The accumulation of acetate increased dramatically upon substrate overloading (data not shown). This might have been the likely reason for the process inhibition. It is important to note that the H_2_ conversion yields in this series of experiments were close to 100 %, which emphasizes the importance of the inoculum quality.

## Discussion

Storage of surplus electricity is a growing demand in renewable energy technology, with the generation of electricity in an inherently fluctuating mode of operation, such as wind and direct solar, gaining a rapidly increasing market share. In a popular strategy, electricity is used to split water and generate H_2_ and O_2_. There are no mature technologies available for handling H_2_ today, and its conversion to CH_4_ therefore seems preferable. In this scheme, electricity is transformed into CH_4_, which is then stored and transported easily via the existing natural gas grids. Chemical methods to reduce CO_2_ with H_2_ have been known for some time and earned the Nobel prize for Paul Sabatier in 1912 [31]. The process requires high temperature, high pressure, and metal catalysts. In alternative electrochemical means of CO_2_ mitigation, electrical energy input is the driving force [3, 9, 30]. Biological systems can solve the same task under mild conditions in an environmentally friendly manner. The life of hydrogenotrophic methanogens, an odd group of Archaea, relies on the same reaction, which is catalyzed by enzymes at ambient temperature and pressure. The biological route of the power-to-gas process, which is here named as power-to-biomethane (P2B), has been recognized and tested in laboratory and scale-up works [19, 22, 24, 32]. These studies have established that microbes are exceedingly efficient catalysts for the P2B process. Hydrogenotrophic methanogens are difficult to cultivate in pure culture, but they are readily available in the mixed culture of effluents from the anaerobic degradation of organic matter, i.e., the fermentation effluent of biogas plants. The rate-limiting step in the work of CH_4_-forming microbial cell factories is the low solubility of H_2_ in the aqueous environment. In previous studies [19, 22, 24, 32], continuously-operating fermentation systems were employed as a rule, which offer several advantageous features for process control and management, but allow short residence time for the injected H_2_ gas.

In our approach, the fed-batch fermentation technique was selected to increase the contact interaction between the gaseous substrate and the biocatalyst methanogens. It was established that an optimal mixing rate has to be upheld in any P2B system in order to facilitate the dissolution of H_2_ into the aqueous phase where the microbes and dissolved CO_2_ reside.

Although CO_2_ is readily soluble in the aqueous medium, it may become an overall limiting factor if removed from the system either by vigorous reaction with H_2_ or by degassing the reactors. Depletion of CO_2_ was accompanied by the elevation of pH, which might be precarious for the activity of hydrogenotrophic methanogens.

CO_2_ is supplied by the biogas-generating process itself [19, 22] or can be provided from outside sources, e.g., flue gas from internal combustion engines. Consumption of the greenhouse gas CO_2_ by the process is an additional benefit of the P2B technology from an environmental point of view. Addition of an organic substrate may revitalize the entire biogas microbial community, which generates additional CO_2_ and thereby stabilizes the pH, but does not facilitate the conversion of H_2_ to CH_4_. A proper feeding routine in the fed-batch system leads to a sustained high rate of CH_4_ formation and the process may operate efficiently for an extended period of time.

### Comparison with previous works

Our approach to improve the P2B principle attempts to counteract the low solubility of H_2_ in the aqueous environment by increasing the contact time of the gas and aqueous phases in a fed-batch fermentation arrangement. This has not been tested earlier.

There are four previous reports available to measure up against this approach. Lee et al. [24] used a fixed-bed reactor, while Reuter [32] developed several versions of a continuous stirred tank reactor (CSTR) design and scaled up the process to an industrial level. Both studies concluded that hydrogenotrophic methanogens in pure or mixed culture were markedly efficient catalysts and converted H_2_ and CO_2_ to CH_4_ in surprisingly high yields and rates. Unfortunately, the published results from those studies contain limited data on process parameters to compare with the fed-batch system examined in the present study.

Two recent papers from the Angelidaki team [19, 22] also used CSTR reactors and reported promising results. Their thoughtfully designed and thoroughly documented reports provided data allowing the assessment with our study. Table 2 summarizes the results.

Besides the use of distinct reactor arrangements and sizes, i.e., fed-batch versus CSTR, several operational parameters differed in those studies from our set-up, e.g., inoculum composition and quality, substrate used for CH_4_ generation, stirring mode and speed. Therefore, only the major tendencies and not the exact values are suitable for a rigorous comparison.

It was found that at high shaking speed the H_2_ conversion process may not be limited by the gas–liquid mass transfer [19] at thermophilic temperature. In our experience, this observation could not be repeated under mesophilic conditions, and above 160 rpm CH_4_ formation was inhibited (Fig. 1). It was concluded that the process in our system was critically limited by the mass transfer of H_2_ at the gas–liquid interface. Hydrogenotrophic methanogens utilized the dissolved H_2_ at a high rate, and therefore a concentration gradient developed between the liquid and gas phases, driving H_2_ into the liquid compartment from the headspace as time advanced. It is likely that the fed-batch operation optimized the condition where the amount of H_2_ transferred into the liquid phase was close to the amount consumed by the microbes. The data presented in Table 2 clearly indicate that this was indeed the case.

In the CSTR work, H_2_ was dosed on the basis of the available CO_2_ from the coupled biogas production [22]. Although significant upgrading of the biogas was achieved, this stipulation limited the rate and amount of H_2_ injection into the system. The goal in these investigations was to achieve maximal H_2_ conversion yield. H_2_ bubbles are difficult to retain in the aqueous system, and diffusers and very low purging rates therefore had to be applied to facilitate the dissolution of H_2_ and its conversion to CH_4_ during the short residence time of the gaseous substrate in the reactor. In the fed-batch configuration, the H_2_ loading rate could be increased to 4 times that of the CSTR operational mode without the loss of H_2_ (Table 2).

In the present study, mesophilic conditions were maintained. Bassani et al. [22] carried out their experiments at 35 and 55 °C under otherwise identical conditions. A significant improvement in CH_4_ formation rate was noted at higher temperature. A similar effect can be expected in the fed-batch system; this will have to be established in future studies. A comparison between our mesophilic data with those obtained at thermophilic temperature indicates a 2.0 [19] and 2.7 [22] times higher CH_4_ production rate from H_2_ in the mesophilic fed-batch reactors as compared with the thermophilic CSTR, respectively.

The mesophilic process performance parameters of Bassani et al. [22] can be compared directly with our results reported under the “Effect of CO_2_ addition” subtitle above. Two sections of stable operation in our experimental period were taken into account, i.e., the initial phase without external CO_2_ addition between days 2 and 28 and the part when stoichiometric CO_2_ and H_2_ were injected daily (days 50–80) (Figs. 5, 6). To make a fair assessment, the residual CH_4_ production in the control reactors (no H_2_ added) should be taken into account.

The control samples in our work started at an unusually low CH_4_/CO_2_ ratio (Table 2), which could be due to the residual biogas potential of the inoculum and the fact that all H_2_ was removed during initial degassing of the reactors. Therefore, the activity of the hydrogenotrophic methanogens was severely restricted until some H_2_ became available from the fermentation of the residual, small amount of biomass. The situation changed dramatically in the reactors receiving H_2_ injections and the system produced bio CH_4_ of high purity, i.e., containing only 17.71 % CO_2_.

There was a 6.5-fold increase in CH_4_ yield from H_2_ in the fed-batch system relative to the mesophilic CSTR experiments if a stoichiometric amount of CO_2_ was added to both systems together with the H_2_ (Table 2). Moreover, the fed-batch system operated at a 4-times higher H_2_ load than the CSTR reactor. The H_2_ consumption was above 90–100 % in both systems, indicating that the reaction was carried out very efficiently in both systems. The CSTR operation mode has its benefits and advantages, but apparently does not help overcome the low H_2_ solubility problem, which seems to be the major bottleneck in the accomplishment of the P2B principle at mesophilic temperature.

**Fig. 9:**
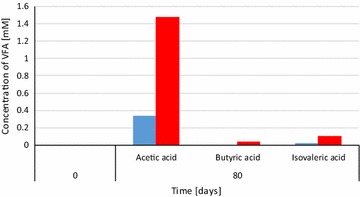
Distribution of volatile fatty acids in the control (*blue*) and (H_2_ + CO_2_)-fed (*red*) reactors. Experimental conditions as in Fig. 5

As an added value, it should be noted that in the fed-batch system a considerable accumulation of acetate takes place without any observable sign of acidosis-related process failure (Fig. 9). The accumulation of acetate was probably due to the inhibition of acetoclastic methanogenesis and syntrophic acetate oxidation [33] by the high H_2_ doses. Acetate is a valuable commodity [30, 34] and, if acetate can be recovered by a suitable technology from the reaction mixture, it would be a useful side-product of the fed-batch fermentation-based P2B technology.

## Conclusions

A general strategy can be proposed on the basis of the results reported above to utilize the microbial community formed in the biogas reactor for the efficient conversion of H_2_ to CH_4_ as part of the P2B principle. Previous studies [19, 22, 24, 32] and the present work unambiguously corroborated that microbiological cell factories are very efficient catalysts to combine H_2_ and CO_2_ to CH_4_, a renewable energy carrier that has already been in use in human practice for many years as fossil natural gas. The suitable microbial community is freely available in the effluent of anaerobic fermentation at the biogas plants operating word-wide in millions of installations at various levels of sophistication.

**Fig. 10:**
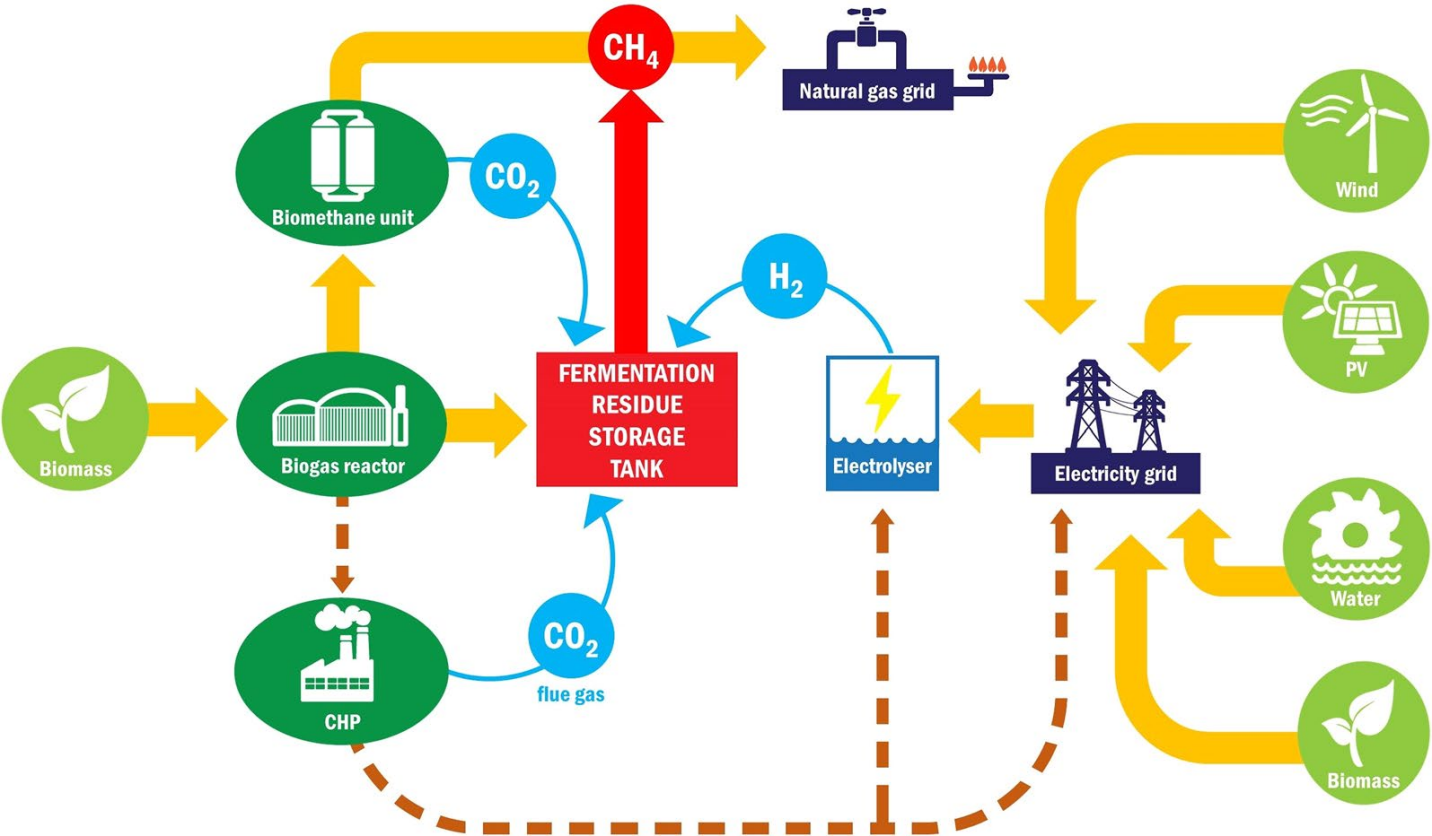
Proposed novel P2B scheme involving the AD fermentation residue storage tank as bio CH_4_ reactor, which converts CO_2_ from biogas or flue gas and H_2_ from electrolysis by renewable electricity

At the center of the projected strategic alliance comprising either of the methods yielding renewable electricity and biogas technology (Fig. 10) are the hydrogenotrophic methanogens present in the biogas effluents. They convert H_2_, which is produced from excess electricity by electrolysis, to CH_4_. BioCH_4_ is relatively easily stored and transported with minor loss in the natural gas grids over large distances and used as energy carrier, biofuel or basic commodity [35], and several technological improvements of bioCH_4_ production [36] have been therefore developed. The proposed novel strategy places biogas technology into the hub of the renewable energy production and utilization network. The biogas effluent reservoir, which forms part of most industrial-scale biogas facilities and stores the digested material until its utilization as organic fertilizer, acquires an entirely new function by becoming a bioreactor to transform green electricity-derived H_2_ into bioCH_4_. The gas to liquid volumetric ratio is lower in industrial-scale effluent reservoirs than the ratio used in our experiments, and installation of a gas recirculation system may therefore be required in the large-scale applications.

The potential economic advantages consequent from the scheme recommended in Fig. 10 are numerous. First, the microbial community present in the biogas effluent can be directly exploited for the efficient conversion of H_2_ and CO_2_ to CH_4_. Second, this biological catalyst is continuously formed at the biogas plants at no additional cost. Third, the microbial community participating in the process is well organized and able to carry out the task under various environmental conditions very efficiently. Fourth, the process is easily manageable, and the microbial community flexibly tolerates the “turn-on” and “turn-off” situations. Fifth, the product is practically pure bioCH_4_ needing no further purification. Sixth, the process also accomplishes a CO_2_ sink and therefore directly contributes to CO_2_ mitigation.

The biogas installations may therefore complement their current operation by becoming bioCH_4_ producers and improve the economy of their technology without substantial additional investments.

## Methods

### The batch fermentation system

The total volume of the reactors was 160 mL (Wheaton glass serum bottle, Z114014 Aldrich). All the samples were run in 3 parallel biological replicates. The reactors routinely contained 40 mL inoculum from the mesophilic industrial biogas plant Zöldforrás Ltd., Szeged, Hungary. The main substrates at Zöldforrás are maize and sweet sorghum silage and pig manure in 80:20 total organic solid ratio. The inoculum was sieved on a 1 mm filter in order to remove the larger particles and was used without further treatment according to the VDI protocol [25]. In each set of experiments, three control reactors containing only the inoculum were included. The calculated amount of solid α-cellulose (C8002 Sigma) was added into the reactors when needed (Table 3). 0.3 g of α-cellulose was routinely added as substrate, as described in the VDI protocol [25]. The daily H_2_ dosage was 0.81 ± 0.16 mmol, unless indicated otherwise. The reactors were sealed with butyl septa and aluminum crimps and were made anaerobic by N_2_ gas exchange of the headspace (5 min). Following the daily gas composition analysis by gas chromatography (GC), the gas phases of the reactors were degassed by purging with N_2_ (Messer nitrogen 4.5) for 5 min and the internal pressure was adjusted to atmospheric level. H_2_ and CO_2_ were injected manually and daily into the gas phase with disposable plastic syringes according to the experimental protocol (Table 3). The amount of the injected gas was verified by GC. The reactors were incubated in a rotary shaker at 37 °C.

**Table 3:** The design of the sample compositions in the various sets of fed-batch reactors

		Series 1: α-cellulose at start				Series 2: α-cellulose at start				Series 3: no α-cellulose				Series 4: α-cellulose weekly			
H_2_ (mmol)^a^		0	0.81	1.43	1.86	0	0.81	1.43	1.86	0	0.81	1.43	1.86	0	0.81	1.43	1.86
	Substrate (g)																
	0.0					X	X				X			X	X		
	0.3	X	X	X	X	X	X							X	X		
	0.6					X	X										
CO_2_ (mL)											5.0^b^						

X indicates the inclusion of the marked component in the reactors. For other experimental conditions see “Methods” section

^a^ Daily injection

^b^ Between day 50 and 80 (see text)

### Organic acid analysis

Samples for organic acid analysis were taken from the liquid phase of the reactors. The samples were centrifuged (13,000 rpm for 10 min,) and the supernatant was filtered through PES centrifugal filter (PES 516-0228, VWR) at 14,000 rpm for 20 min. The concentrations of volatile organic acids were measured with HPLC (Hitachi LaChrome Elite) equipped with refractive index detector L2490. The separation was performed on an ICSep ICE-COREGEL—64H column. The temperature of the column and detector was 50 and 41 °C, respectively. The eluent was 0.01 M H_2_SO_4_ (0.8 mL min^−1^).

### Gas composition analysis

The gas composition of the reactor headspace was measured every day by GC. The CH_4_ and H_2_ contents were determined with an Agilent 6890 N GC (Agilent Technologies) equipped with an HP Molesive 5 Å (30 m × 0.53 mm × 25 µm) column and a TCD detector. The temperature of the injector was 150 °C and application was made in split mode 0.2:1. The column temperature was maintained at 60 °C. The carrier gas was Linde HQ argon 5.0, with the flow rate set at 16.8 mL min^−1^.

The amount of CO_2_ was determined with a Shimadzu GC 2010 (Shimadzu Corporation) equipped with a TCD detector and a HP PlotQ (30 m × 0.5 mm × 40 µm) column. The chromatograph was applied in split injection mode (rate 0.5:1). The temperature of the inlet was 200 °C. The column and the detector temperature were maintained at 90 and 150 °C, respectively. The applied carrier gas was Messer nitrogen 4.5 at 8.4 mL min^−1^. The samples were injected with the help of a gastight microsyringe (Hamilton). The conversion efficiency of H_2_ to CH_4_ was calculated by the modified theoretical equation [15].

$$ \eta = \frac{{\left( {{\text{r}}_{{CH_{4} A}} - {\text{r}}_{{CH_{4} B}} } \right)}}{{\left( {{\text{r}}_{{H_{2} A}} - {\text{r}}_{{H_{2} D}} } \right) \times 4}} \times 100 $$

where “A” is the experimental reactor and η = conversion efficiency of H_2_ to CH_4_ (%) ${\text{r}}_{{CH_{4} A}}$ = CH_4_ production of reactor A (mL L^−1^ h^−1^) ${\text{r}}_{{CH_{4} B}}$ = CH_4_ production of control reactor (mL L^−1^ h^−1^) ${\text{r}}_{{H_{2} A}}$ = the added amount of H_2_ to reactor A (mL L^−1^ h^−1^) ${\text{r}}_{{H_{2} D}}$ = the residual amount of H_2_ in reactor A (mL L^−1^ h^−1^).

## Determination of fermentation parameters

oDM: The organic dry matter content was quantified by drying the biomass at 105 °C overnight and weighing the residue, giving the dry mass content. Further heating of this residue at 550 °C provided the organic dry matter (oDM) content.

pH: The value of the pH was measured with a Radelkis OP-211/2 equipped with an OP-0808P pH electrode immediately after the daily GC analysis.

## Abbreviations

CSTR: continuous stirred tank reactor
P2B: power to biomethane concept
Rpm: revolution per minute
GC: gas chromatograph
HPLC: high-pressure liquid chromatography
TCD: thermal conductivity detector
oDM: organic dry matter content
